# Current Methods for Automated Filtering of Multiple Sequence Alignments Frequently Worsen Single-Gene Phylogenetic Inference

**DOI:** 10.1093/sysbio/syv033

**Published:** 2015-06-01

**Authors:** Ge Tan, Matthieu Muffato, Christian Ledergerber, Javier Herrero, Nick Goldman, Manuel Gil, Christophe Dessimoz

**Affiliations:** ^1^Department of Computer Science, ETH Zurich, Universitätstr. 6, 8092 Zurich, Switzerland,; ^2^Department of Molecular Sciences, Institute of Clinical Sciences, Faculty of Medicine, Imperial College London, London, UK;; ^3^MRC Clinical Sciences Centre, London W12 0NN, UK;; ^4^European Molecular Biology Laboratory, European Bioinformatics Institute, Hinxton, Cambridge, CB10 1SD, UK;; ^5^University College London, Gower St, London WC1E 6BT, UK; and; ^6^Institute of Molecular Life Sciences, University of Zurich, Winterthurerstr. 190 , 8057 Zurich, Switzerland; and; ^7^Swiss Institute of Bioinformatics, Universitätstr. 6, 8092 Zurich, Switzerland

**Keywords:** alignment filtering, alignment trimming, molecular phylogeny, multiple sequence alignment, phylogeny, phylogenetic inference, phylogenetics

## Abstract

Phylogenetic inference is generally performed on the basis of multiple sequence alignments (MSA). Because errors in an alignment can lead to errors in tree estimation, there is a strong interest in identifying and removing unreliable parts of the alignment. In recent years several automated filtering approaches have been proposed, but despite their popularity, a systematic and comprehensive comparison of different alignment filtering methods on real data has been lacking. Here, we extend and apply recently introduced phylogenetic tests of alignment accuracy on a large number of gene families and contrast the performance of unfiltered versus filtered alignments in the context of single-gene phylogeny reconstruction. Based on multiple genome-wide empirical and simulated data sets, we show that the trees obtained from filtered MSAs are on average worse than those obtained from unfiltered MSAs. Furthermore, alignment filtering often leads to an increase in the proportion of well-supported branches that are actually wrong. We confirm that our findings hold for a wide range of parameters and methods. Although our results suggest that light filtering (up to 20% of alignment positions) has little impact on tree accuracy and may save some computation time, contrary to widespread practice, we do not generally recommend the use of current alignment filtering methods for phylogenetic inference. By providing a way to rigorously and systematically measure the impact of filtering on alignments, the methodology set forth here will guide the development of better filtering algorithms.

## Introduction

Phylogenetic reconstruction pervades computational and evolutionary biology; it is thus important to be able to compute accurate phylogenetic trees. While it is possible to infer phylogenetic trees directly from sequence data (Jun et al. 2009), the most common inference methods build on multiple sequence alignments (MSA) (Felsenstein 1989; Ronquist and Huelsenbeck 2003; Stamatakis 2006; Guindon et al. 2010). Hence, the accuracy of phylogenetic trees inherently depends on the accuracy of the underlying MSA.

Given a set of sequences, an ideal MSA identifies homologous characters, that is, characters having common ancestry. However, computing such an MSA can be challenging. While most alignment programs will correctly identify and align highly conserved regions, regions containing a large number of insertions and/or deletions are typically less reliable. Such unreliable sections and erroneously aligned residues can negatively affect downstream analyses, such as tree inference (Lunter et al. 2008; Wong et al. 2008; Dessimoz and Gil 2010).

Filtering—that is, removing unreliable columns before tree reconstruction—has been promoted as a way to increase the signal to noise ratio of MSAs (Talavera and Castresana 2007). Despite evidence that correlation among sites can affect phylogenetic inference (Nasrallah et al. 2011), almost all commonly used phylogenetic inference methods—be they of the likelihood, distance, or parsimony paradigms—are based on models assuming site independence and hence can be applied to any subset of the alignment columns. The difficulty of filtering alignments, however, lies in effectively detecting unreliable columns without removing phylogenetically informative sites.

In recent years, a number of software packages aimed at detecting unreliable alignment columns have been published. They take a wide variety of different approaches, from ad hoc rules based on substitution patterns (Castresana 2000; Capella-Gutiérrez et al. 2009) to more rigorous approaches based on models of phylogenetically informative versus uninformative sites (Dress et al. 2008; Kück et al. 2010). In the past few years, several simulation studies have investigated the impact of filtering sequence alignments for inference of sites under positive selection (Fletcher and Yang 2010; Jordan and Goldman 2012; Privman et al. 2012). But in the context of phylogenetic inference, a systematic and comprehensive comparison of different alignment filtering methods has been missing. In particular, the performance comparisons provided in the articles presenting each filtering method are based on very small data sets (Castresana 2000; Dress et al. 2008; Kück et al. 2010) and/or simulated data, which may lack realism and provide limited empirical value (Talavera and Castresana 2007; Capella-Gutiérrez et al. 2009; Kück et al. 2010).

Recently, Dessimoz and Gil (2010) introduced phylogeny-based tests of alignment accuracy, which use large samples of empirical data. Here, we present four tests to assess the effect of alignment filtering on phylogenetic reconstruction. By applying these tests on large empirical and simulated gene sets, we show that current automated-filtering approaches do not lead to better reconstruction of single-locus trees. Note that this study focuses on the accuracy of tree topology (i.e., branching order), not branch lengths, and that we do not consider filtering approaches that mask individual sequences or characters.

## Methods

We first review the filtering methods considered in this study. Next, we introduce the four main assessment methods used to support our main conclusions (species discordance test, minimum duplication test, Ensembl pipeline, and simulation). The remainder of this section provides a detailed exposition of the empirical and simulated data underpinning our analyses, the alignment and tree methods, and the alignment accuracy measures used in the study.

### Filtering Methods Included in This Study

Filtering methods take a variety of mathematical and heuristic approaches. Those considered here all have in common that they are fully automated and they remove entire columns of the alignment. Our (non-exhaustive) selection includes a broad range of software packages that distinguish themselves by popularity or originality (Table 1). One of the first methods introduced was Gblocks (v0.91b) (Castresana 2000; Talavera and Castresana 2007). In the first step, Gblocks classifies each column as nonconserved, conserved, or highly conserved depending on the number of identical residues in this column and on the presence/absence of gaps. Based on this classification, contiguous stretches of nonconserved positions are removed. Further filtering is applied such that every block is flanked by highly conserved positions, serving as high confidence anchors. Next, short blocks (≤15 columns) are removed; finally, all gappy positions and their adjacent nonconserved residues are removed. This is followed by another round of removal of short (≤10 columns) blocks. In the analyses below, we included both default and relaxed settings (“Minimum Number of Sequences for a Flank Position” = 9, “Maximum Number of Contiguous Nonconserved Positions” = 10, “Minimum Length of a Block” = 5, and “Allowed Gap Positions” = “With Half”), as described by the authors (Talavera and Castresana 2007).

**Table 1.** Overview of filtering methods considered in this study

Filtering methods	Type of “undesirable” sites filtered out by the method	Accounts for tree structure?	Uses a substitution matrix or model of evolution?	Adapts parameters for particular data sets?	References
**Gblocks**	Gap-rich and variable sites	No	No	No	Talavera and Castresana (2007)
**TrimAl**	Gap-rich and variable sites	No	Yes	Yes	Capella-Gutiérrez et al. (2009)
**Noisy**	Homoplastic sites	In part	No	No	Dress et al. (2008)
**Aliscore**	Random-like sites	No	Indirectly	No	Kück et al. (2010)
**BMGE**	High entropy sites	No	Yes	No	Criscuolo and Gribaldo (2010)
**Zorro**	Sites with low posterior	Yes	Yes	No	Wu et al. (2012)
**Guidance**	Sites sensitive to the alignment guide tree	Yes	Indirectly	No	Penn et al. (2010)

Of the other methods considered here, TrimAl v1.2 (Capella-Gutiérrez et al. 2009) is conceptually closest to Gblocks. Columns are removed according to a threshold value on their score. The score has two main components: a gap score component (% of sequences containing a gap, analogous to the presence/absence of gaps in Gblocks), and a residue similarity score component (using a model of substitution with the aim of capturing more information than just the proportion of identical residues considered in Gblocks). Optionally, TrimAl can also compute a consistency score component among several provided alignments. The main difference with Gblocks, however, is that TrimAl can not only trim according to some user-defined thresholds but also has a number of built-in heuristics which allow automatic per-alignment selection of those thresholds. The three heuristics tested in this study are gappyout, strict, and automated1. The gappyout heuristic sets the gap threshold parameter—the minimum proportion of sites in a column—at the point where the variation of the proportion of alignment removed is greatest (i.e., point of greatest gradient). The strict heuristic uses the same approach but trims the alignment further based on an automatically selected similarity score threshold. The automated1 option chooses between gappyout and strict based on a decision tree optimized on a benchmark (Capella-Gutiérrez et al. 2009).

Whereas Gblocks and TrimAl are based on sitewise summary statistics of MSAs, other methods are based on mathematical models. Noisy (v.1.5.11) tries to infer columns that are phylogenetically uninformative by assessing the degree of homoplastic sites compared to random columns (Dress et al. 2008). Rather than assessing character compatibility on trees, they look at the distribution of column characters on circular orderings of the taxa, a more general structure (Makarenkov and Leclerc 1997; Semple and Steel 2004). In this way, they can compute a character compatibility score without assuming a particular tree topology. Of note, its authors caution that Noisy needs an alignment of at least 15 sequences to perform well (Dress et al. 2008).

Aliscore v.1.0 (Kück et al. 2010) assesses the randomness of a MSA by considering all the induced pairwise alignments separately, using a sliding window. The alignment within a given window is considered random if the score of the alignment within that window is not better than the 95th percentile score of random pairwise alignments. Random alignments are sampled according to background character frequencies estimated from the neighborhood of the sliding window. The decision for a particular residue in the alignment is based on the majority of all the windows containing that residue. Finally, a position in the alignment is considered random if the majority of the induced pairwise alignments are random at that position.

Block Mapping and Gathering with Entropy (BMGE) (Criscuolo and Gribaldo 2010) attempts to identify runs of unexpectedly variable alignment columns by computing an entropy measure over a sliding window and removing columns that fall above a certain cutoff. The entropy measure takes into account the similarity of DNA or amino acid characters corresponding to a fixed level of divergence set by the user. In doing so, it ignores any underlying tree structure and thus treats each character as an independent observation. The software package also provides a way of filtering individual characters, but this variant lies outside the scope of this study.

Zorro (previously also known as Probmask; Wu et al. 2012) estimates a confidence score for each column and removes columns that fall below a certain threshold, which the user can specify. In brief, the confidence score for a particular column is obtained from the weighted average of the posterior probabilities that each pair of characters in a column are aligned. The probabilities are computed based on a single-pair hidden Markov model, with parameters estimated from all pairs of sequences. The weights, which are designed to account for the correlation among the pairs, are computed based on a guide tree estimated from the alignment.

Finally, Guidance (Penn et al. 2010) was developed under the premise that errors in the guide tree used for the alignment process strongly perturb the inferred alignment. Guidance estimates the reliability of alignment columns from their respective frequencies in “resampled” MSAs obtained based on guide tree bootstrap replicates (100 replicates by default). Thus, uniquely among the filtering methods considered here, Guidance requires knowledge of the aligner that was used to produce the MSA to be filtered. Furthermore, because of the need to compute a new alignment for each guide tree bootstrap replicate, Guidance is computationally very costly compared to the other methods.

### Tests to Assess Filtering Methods

We assessed the performance of filtering methods using four kinds of tests. The first two are extensions of recently introduced phylogenetic tests of alignment accuracy (Dessimoz and Gil 2010). The *species tree discordance test* uses orthologous sequences from species with an undisputed branching order and exploits the fact that orthologs, by definition (Fitch 1970), should conform to the species tree. Thus, a particular filter is beneficial if it leads to trees that are more similar to the species tree than trees from unfiltered alignments (i.e., shows a decrease in the fraction of incorrect gene tree splits), and detrimental otherwise (i.e., shows an increase in the fraction of incorrect gene tree splits).

The *minimum duplication test*, uses homologous sequences and assumes a parsimonious duplication history to be most likely (Dessimoz and Gil 2010). The central assumption is that all others being equal, more accurate gene trees tend to require fewer duplications (and thus also losses) to explain the history of gene families. This assumption lies at the heart of most gene/species tree reconciliation methods (Goodman et al. 1979; Zmasek and Eddy 2001).

**Figure 1.**
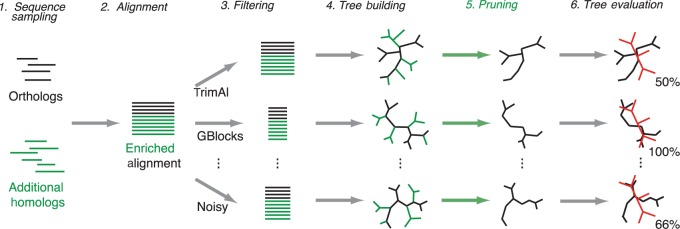
Schematic of the species tree discordance test used to evaluate filtering methods. The grey (green in online version) elements indicate extra steps involved in the *enriched* version of the test. The tests sample sets of orthologs with an undisputed phylogeny (black sequences). The enriched test adds homologous sequences with unknown branching order (green in online version). The input sequences are aligned and then filtered by the different filtering methods. The filtered alignments are evaluated by reconstructing trees from them, which are compared with the reference topology (red in online version). In the enriched test, all additional sequences are removed from the tree and what remains (subtree relating the orthologous sequences) is compared to the reference topology. The unfiltered alignment is evaluated in the same way. All others being equal, the relative performance of the filtering methods can be assessed by their average congruence with the reference topology over a large number of input problems.

Because the species tree discordance test requires trusted, fully resolved topologies, it tends to be restricted to small sets of sequences. We chose to be very cautious and to use sequences from small six-species sets for which the species tree is incontrovertible (see section “Methods”). The downside of this approach is that findings based on such six-sequence alignments might not generalize well to larger alignments. To test larger alignments, one could use larger trusted topologies, but larger topologies can be more difficult to defend. Instead, we have developed a new test variant, which we call the “enriched species tree discordance test” (Fig. 1). In this variant, the original six orthologs are augmented with a number of homologous sequences. The combined set is then aligned and the various filtering methods are applied to the resulting MSAs. Subsequently, trees are estimated from the filtered and unfiltered MSAs. To evaluate the resulting trees, all leaves corresponding to additional homologs are pruned, leaving the subtree consisting of the original six taxa only, whose topology can be compared to the reference species topology. If available, larger trusted topologies would be preferable, because they would allow us to measure all topological differences in the augmented trees and thereby confer a higher statistical power to the test. But as long as the systematic improvement or worsening also affects the placement of the original sequences, this protocol makes it possible to compare the quality of larger alignments and trees without requiring the trusted topology to be larger (i.e., better resolved).

To assess the impact of branch support on the analyses, we modified the enriched species discordance test as follows. In the tree inference step (Fig. 1, step 4), we computed approximate Bayesian posterior (Anisimova et al. 2011) as the measure of support for each branch in the enriched trees (reconstructed with or without filtering). In the pruning stage (Fig. 1, step 5), pruning sequences leads to branches getting merged. As support value for merged branches (branches corresponding to paths in the enriched gene trees), we used the maximum support of all the merged branches, which is a conservative estimate of the actual support. We then disregarded all branches with support below a set threshold. The remaining branches were counted as true positives if they were present in the reference tree and as false positives otherwise. Branches only present in the reference trees were counted as false negatives. Furthermore, to assess different level of stringencies, we also repeat the entire analysis for different support threshold values.

The third kind of filtering assessment method used in this study is based on the Ensembl Compara data and pipeline, which infers gene trees reconciled with a reference species tree (i.e., gene trees whose inner nodes are labeled as speciation or duplication events) (Vilella et al. 2009). As Vilella *et al.* emphasize in their article, some resulting reconciled trees contain very poorly supported duplication nodes (called “dubious” nodes): these nodes are followed by differential gene losses, as opposed to strongly supported duplications, where both duplicated genes remain in most of the subtrees. To gauge the impact of alignment filtering on the reconciled gene trees generated by the Ensembl pipeline, we use the average number of losses per branch over all trees as indicator. If filtering leads to the average number of losses per branch significantly decreasing, the parsimony principle suggests that gene tree inference and reconciliation has improved and therefore alignment filtering is on the whole beneficial. If the average number of losses per branch increases, the opposite holds. The criterion used in this test is conceptually similar to the minimum duplication test, but the two were implemented independently and differ in several minor points: contrary to the minimum duplication test, this one only considers a single-gene tree rooting (inferred in the tree reconciliation), counts losses instead of duplications (both are correlated but not exactly so), and uses a reference species tree in the reconciliation step.

Fourth, in addition to these various sets of empirical data, we also evaluated the effect of alignment filtering on simulated data. We used the species tree discordance test, using the true tree as reference. Because the correct alignment is known with certainty in simulated data, we were also able to directly measure the effect of filtering on the alignment itself.

Besides these four approaches, we note that other classes of alignment benchmarking techniques could conceivably be used, such as benchmarks based on protein structure conservation; however, these other approaches also have their assumptions and shortcomings. We refer the interested reader to a recent review on this topic (Iantorno et al. 2014).

### Data

#### Empirical data

The species tree discordance test and its enriched version were executed on three taxonomic ranges: fungi, eukaryotes, and bacteria. A total of 10,999 sets of six orthologs were sampled from the September 2008 Orthologous MAtrix (OMA) database release (Altenhoff et al. 2011) relying on to the same species with undisputed topology as in Dessimoz and Gil (2010) (Supplementary materials available on Dryad at http://dx.doi.org/10.5061/dryad.pc5j0). Additional homologs were automatically collected from the SwissProt database via NCBI BLAST using the script Mafft-Homologs (Katoh et al. 2005), with a threshold E-value of $10^{-10}$. Note that only the aligned (homologous) portion of such additional sequences is considered, except for one control experiment. When Mafft-Homologs returned fewer than 24 sequences, they were all kept; otherwise, 24 sequences were randomly selected, resulting in problem sets of up to 30 sequences (∼26 on average) for the enriched test.

For the minimum duplication test, we retrieved groups of up to 60 homologs (∼36 on average) from eukaryotic and fungal genomes. Both the species tree discordance test and the minimum duplication test were run once on amino acid and once on nucleotide data.

The setup resulted in highly diverse conditions, which have been characterized in terms of percentage sequence identity, sequence length, and number of sequences (Supplementary Figs. 2–4 available on Dryad at http://dx.doi.org/10.5061/dryad.pc5j0). The enrichment led, for all three phyla, on average to shorter sequences than the original 6 orthologous sequences (by about 100 amino acids). This is explained by the local matching of BLAST (recall that we only retrieve the aligned portion of additional homologs). The average percent identity decreased slightly for fungi and eukaryotes (by 0.25% and 0.48%, respectively), and increased for bacteria by about 2%. Alignments and trees for all data sets are available as raw data on Dryad at http://dx.doi.org/10.5061/dryad.pc5j0.

The tests based on Ensembl data use version 66 (February 2012), which contains 19,491 homologous clusters (and their corresponding MSAs/trees) containing 969,577 protein-coding genes from 57 species. The distribution of the cluster sizes is uneven (Supplementary Fig. 5 available on Dryad at http://dx.doi.org/10.5061/dryad.pc5j0). Since the tests are based on variations in rooted tree topology (see above), we considered clusters with at least four genes and two supporting species (11,321). Of these, 11,231 could be processed with all filtering methods and parameters such that there was at least one column in the resulting alignment. The resulting data set covered 898,138 genes (92.6%) of the initial gene set.

Of note, dubious nodes in the Ensembl data set are not concentrated in places with documented whole-genome duplications and/or bursts of segmental duplications, but rather in places with rapid species radiation (Supplementary Fig. 6 available on Dryad at http://dx.doi.org/10.5061/dryad.pc5j0), which suggests that the majority of these dubious nodes are due to limitations in the inference process and not bona fide differential gene losses that would be overly penalized by the parsimony criterion.

To investigate the differences with respect to cluster size (and thus number of species per alignment), we defined subsets depending on cluster size with respect to the total number of species *ns* (=57): “small MSAs” cover fewer than *ns*/2 species and contain fewer than *ns*/2 genes; “medium MSAs” cover at least *ns*/2 species and contain between *ns*/2 and 3*ns*/2 genes; “large MSAs” cover at least *ns*/2 species and contain more than 3*ns*/2 genes. These categories are disjoint and collectively cover 97.4% of all the MSAs we could process (Supplementary Table 1 available on Dryad at http://dx.doi.org/10.5061/dryad.pc5j0).

In each analysis reported here, to ensure that results are comparable across all methods tested, we only considered alignments that lead to trees for all filtering methods considered (in a small minority of the cases, the most aggressive filtering methods removed all columns of the input alignment).

#### Simulated data

Two sets of 500 30-sequence MSAs were simulated using Artificial Life Framework (ALF) (Dalquen et al. 2012). In both cases, the sequence length was drawn from a Gamma distribution (with parameters k=2.78,θ=133.81). Sequences were evolved along 30-taxa birth–death trees (with parameters λ=10μ) scaled such that the distance from root to deepest branch was either 250 point accepted mutation (PAM) units for the main data set, or 100 PAM for the control data set. For both data sets, characters were substituted according to WAG substitution matrices (Whelan and Goldman 2001), and insertions and deletions were applied at a rate of 0.0001 event/PAM/site, with length following a Zipfian distribution with exponent 1.821 truncated to at most 50 characters (default ALF parameters).

### MSA Methods

Jordan and Goldman (2012) have shown that, at least in the context of sitewise detection of positive selection, there is an interaction between the aligner and the filtering method chosen. Thus, to test the filtering methods on a range of aligners, we computed initial alignments with Mafft 6.843 (Katoh et al. 2005), Prank 100802 (Löytynoja and Goldman 2005), ClustalW 2.0.10 (Thompson et al. 1994) and T-Coffee v.5.72 (by default) and T-Coffee v.10.00 (only where specified) (Notredame et al. 2000). The argument of Mafft was “*–retree 2*”. For Prank, the option “*-F*” was used. ClustalW and T-Coffee were used with their default parameters.

The tests on Ensembl data were based on the regular Ensembl Compara multiple alignments which, depending on the cluster size, are computed with either Mafft (Katoh et al. 2005) or M-coffee (Wallace et al. 2006). Mafft is used with the parameter auto and M-coffee is called to combine alignments from Mafft, Muscle (Edgar 2004), Kalign (Lassmann and Sonnhammer 2005) and T-Coffee (Notredame et al. 2000).

### Tree Reconstruction Methods

Phylogenetic trees were reconstructed by two methods: maximum likelihood and a least-squares distance approach. PhyML v3.0 (Guindon et al. 2010) was run with either WAG substitution matrices (Whelan and Goldman 2001) for amino acid data or General Time-Reversible (GTR) for nucleotide data, with gamma-distributed rates among sites (using discrete gamma approximation with four categories), an estimated proportion of invariable sites (i.e., WAG+Γ + I or GTR+Γ + I), and nearest neighbor interchange as topological search strategy. As control, the *MinSquareTree* function in Darwin (Gonnet et al. 2000) was used to reconstruct variance-weighted least-squares trees. This optimizes topologies using nearest neighbor interchanges around four- and five-taxa configurations.

The tests on Ensembl data used TreeBeST, just as in the regular Ensembl Compara pipeline (Vilella et al. 2009). In essence, TreeBeST performs gene tree inference and reconciliation with a reference species tree obtained from the NCBI taxonomy database (Sayers et al. 2010). The reconciliation is optimized over gene trees built from five different models—a species tree aware version of PhyML (http://sourceforge.net/projects/treesoft/; (Guindon et al. 2010)) with the HKY (Hasegawa et al. 1985) and WAG models (Whelan and Goldman 2001), as well as Neighbor-Joining (Saitou and Nei 1987) over *dn*, *ds* and *p*-distances—minimizing the number of duplications and gene losses.

### Alignment Comparison Measures

To assess the accuracy of alignments performed on simulated data, for which we know the true alignment, we computed the precision and recall of alignments using the sum-of-pair measure (Sauder et al. 2000). For precision, we compute the fraction of residue pairs in the filtered alignment that are present in the reference alignment:

$$precision=\frac{1}{\left(\begin{array}{c} N\\ 2 \end{array}\right)}\sum_{i,j\;\text{s}.\text{t}.\;i<j}f_{D}(S_{i},S_{j})$$

where N is the number of sequences in the alignment, and $f_{D}(S_{i},S_{j})$ is the number of correctly aligned residues between sequences $S_{i}$ and $S_{j}$ in the test alignment divided by the number of aligned residues between sequences $S_{i}$ and $S_{j}$ in the test alignment (Sauder et al. 2000).

For recall, we compute the fraction of residue pairs in the true alignment that are present in the filtered alignment:

$$recall=\frac{1}{\left(\begin{array}{c} N\\ 2 \end{array}\right)}\sum_{i,j\;\text{s}.\text{t}.\;i<j}f_{M}(S_{i},S_{j})$$

where N is the number of sequences in the alignment, and $f_{M}(S_{i},S_{j})$ is the number of correctly aligned residues between sequences $S_{i}$ and $S_{j}$ in the test alignment divided by the number of aligned residues between sequences $S_{i}$ and $S_{j}$ in the true alignment (Sauder et al. 2000).

Alternatively, we also used precision and recall based on the more the simple “true column” measure as described in Thompson et al. (2005).

## Results

We first present an in-depth assessment of filtering methods based on the enriched species discordance test. The analyses consistently indicate that on average and across all data sets, trees reconstructed from filtered alignments are generally worse than those obtained from unfiltered alignments. Next, we show that our main findings hold when controlling for numerous potential confounding factors (MSA/tree inference programs, sequence length, sequence divergence, alignment “gappiness”), and when considering the minimum duplication test. Finally, we show that our conclusions are also corroborated by analyses based on the Ensembl Compara pipeline and data, and by analyses on simulated data.

In aggregate, the main analyses reported here required computing over 540,000 alignments of up to 60 sequences and over 11.1M phylogenetic trees, for a total computational cost of over 1.26M CPU hours. For the analyses on Ensembl Compara data, 900,000 trees of up to 400 sequences were computed for a total runtime of 150,000 CPU hours.

### Results on the Enriched Species Discordance Test

We first compared the filtering methods on the enriched species tree discordance test (Fig. 1), based on Prank alignment (Löytynoja and Goldman 2005). We performed separate analyses on protein-coding sequences in three broad taxonomic ranges (fungi, eukaryotes, bacteria), both at amino acid and nucleotide levels. Sequences were aligned by Prank and phylogenetic trees were inferred by maximum likelihood (model WAG + Γ + I for protein sequences and model GTR + Γ + I for nucleotides). Note that the data sets and methods are described in detail in the “*Methods*” section, and that controls using other alignment programs, testing strategies, and data follow after we have presented our main results.

#### Alignment filtering does not improve phylogenetic tree inference

**Figure 2.**
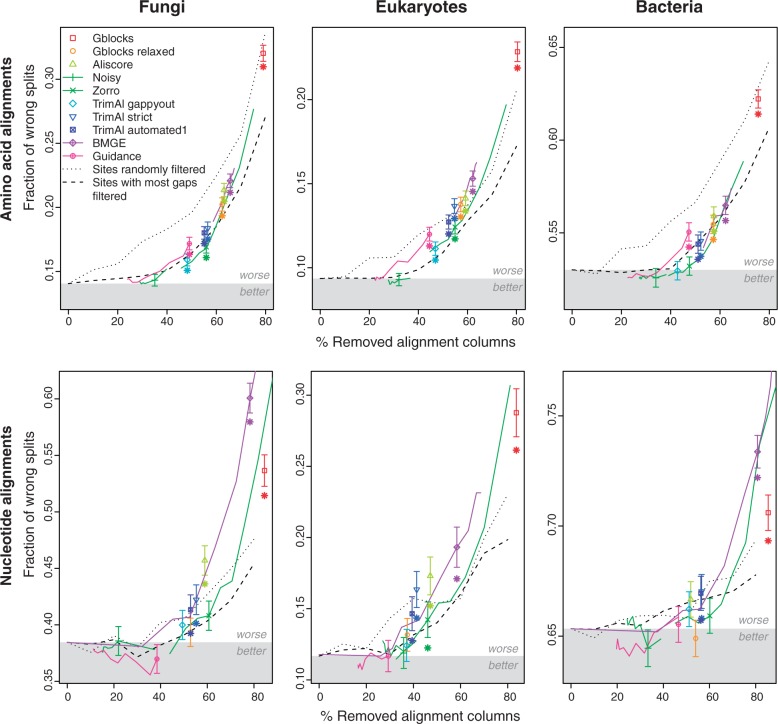
Alignment filtering generally yields poorer phylogenetic trees. Depicted here are results with the enriched species tree discordance test on amino acid (top) and nucleotide (bottom) alignments from three taxonomic ranges. The measure of error is the average RF distance between the reference trees and trees reconstructed from Prank + F alignments filtered by the various approaches. Trees were reconstructed using PhyML. Filtered alignments improving over unfiltered alignment fall in the gray region. The two dotted lines correspond to results obtained with two simplistic filtering methods (see main text). Points correspond to default parameters. Colored lines are linear interpolations between additional points obtained with non-default parameters (not available for all methods). Error bars indicate the standard error of the mean. If a filtering method with default parameters yields significantly different (two-sided Wilcoxon test, α=0.01) results from unfiltered alignments, a star is displayed below the corresponding point. Note that no multiple testing correction were applied.

Overall, we found that tree inference does not generally improve after alignment filtering (Fig. 2). With amino acid alignments, none of the filtering methods resulted in significant improvement (two-sided Wilcoxon test of paired samples, P≥0.01); on the contrary, most of them decreased tree reconstruction accuracy, at times strongly so (Fig. 2, top). We had previously observed that amino acid alignments tend to be more accurate than nucleotide ones (Dessimoz and Gil 2010); one could thus expect filtering methods to have more opportunities to improve nucleotide alignments. In the present study, filtering fared slightly better on nucleotide alignments indeed, yet no combination showed significant improvements over unfiltered alignment (two-sided Wilcoxon test, P≥0.01); instead, most cases were either insignificant or significantly worse than unfiltered alignments (Fig. 2, bottom).

#### Parameter optimization helps, but not enough to make filtering generally worthwhile

The poor performance of some of the filtering methods can be partly explained by inadequate default parameter values. For instance, consistent with previous observations (Talavera and Castresana 2007; Criscuolo and Gribaldo 2010), the much improved performance of Gblocks “relaxed” compared to default settings suggests that its default parameters are too strict (Fig. 2). One of the largest improvements afforded by parameter optimization was with Guidance: lowering the colCutoff parameter to 0.2 substantially reduced the error rate of reconstructed trees relative to the default parameter (0.93; Supplementary Fig. 7 available on Dryad at http://dx.doi.org/10.5061/dryad.pc5j0); two-sided Wilcoxon test, $P<10^{-17}$). With optimized parameters, Guidance was no longer detrimental; on nucleotide data it was even marginally beneficial. Likewise, lowering Zorro's threshold parameter (from 4 to 1 or 2) noticeably improved the quality of the trees over the default (Supplementary Fig. 8 available on Dryad at http://dx.doi.org/10.5061/dryad.pc5j0); two-sides Wilcoxon test, $P<10^{-117}$) and so did optimising the choice of BMGE's similarity matrix (from BLOSUM62 down to BLOSUM30; Supplementary Fig. 9 available on Dryad at http://dx.doi.org/10.5061/dryad.pc5j0), $P<10^{-80}$). For Noisy, the default parameter (0.8) proved to be closer to optimal, but lowering it (to 0.3–0.4) yielded better results overall (Supplementary Fig. 10 available on Dryad at http://dx.doi.org/10.5061/dryad.pc5j0); $P=1.12\times 10^{-4}$). Finally, note that we could not optimize parameters for all methods, because some of them have so many parameters that optimization becomes impractical (Gblocks, Aliscore, TrimAl).

Even with optimized parameters, most filtering methods did not yield better trees than unfiltered alignments, especially with amino acid alignments (Fig. 2, colored lines).

#### Filtering methods hardly outperform simple baselines

To put the performance of filtering methods into perspective, we sought to compare the filtering methods to two trivial approaches. The first entails randomly discarding x% of all alignment columns; the second entails removing the x% of columns with most gap characters. We plotted the percentage wrong splits as a function of the percentage of removed sites, and compared the filtering methods against these two baselines (Fig. 2, dotted lines). Compared to unfiltered alignment, unsurprisingly, discarding random alignment columns consistently worsened the trees (two-sided Wilcoxon test, P<0.01 beyond 20% of columns removed). Albeit to a lesser extent, so did removing the most gap-rich columns (two-sided Wilcoxon test, P<0.01 beyond 40% of columns removed). But worryingly, relative to these baselines, the performance of most filtering methods proved to be mediocre: only in a minority of the cases did they perform better than both baselines; on some combinations of data sets and methods, the results turned out to be even worse than with random filtering.

#### More aggressive filtering generally results in poorer trees

Finally, the results show that there is a correlation between the percentage of sites removed and the tree reconstruction error rate, suggesting that more aggressive filtering tends to yield worse trees (Fig. 2; Spearman correlation: 0.93–0.96, $P<1.4\times 10^{-4}$ for amino acid alignments and 0.77–0.96, P<0.014 for nucleotide alignments). This is consistent with our observations above on parameter tuning, where the best performances are usually obtained with parameter values resulting in fewer sites removed.

It should nevertheless be said that the relation between sites removed and tree performance is not linear: for up to 20–30% of the sites removed, trees did not noticeably deteriorate. Beyond this point, trees deteriorated rapidly. Given that computational and space complexity of typical tree inference methods scale roughly linearly with the alignment length, this result suggests that a modest degree of filtering can be warranted to reduce computational and memory costs.

#### Filtering not only increases the proportion of unresolved branches, but also often increases the proportion of well-supported branches that are wrong

Until this point, we have treated inferred trees without consideration of branch support. Yet in principle, our results described so far could be consistent with alignment filtering providing a way to decrease the proportion of incorrect branches that have high support (and thus mislead) at the cost of increasing the proportion of unresolved branches (which are merely uninformative). These types of errors can be thought of as false positive and false negative, respectively (see section “Methods”).

**Figure 3.**
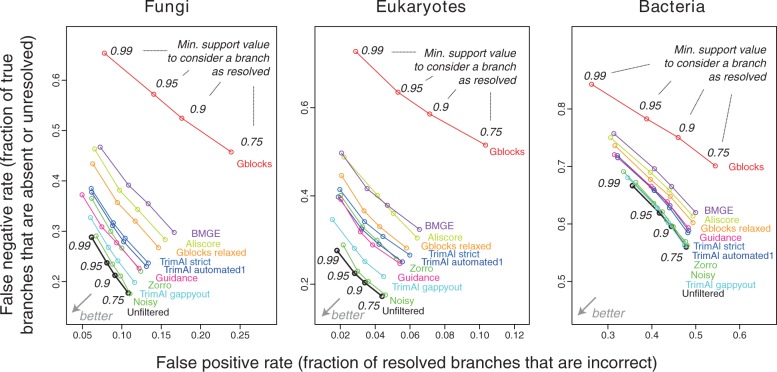
Filtering not only increases the fraction of branches that are unresolved, but also often increases the fraction of resolved branches that are incorrect. Using approximate Bayesian posterior as the branch support measure (Anisimova et al. 2011), we considered branches below particular branch support values as unresolved (cutoff values in italics) in the enriched species discordance test on amino acid sequences.

We decomposed the effect of the various filtering methods on the false positive and false negative rates, using the enriched species discordance test and taking the approximate Bayesian posterior (Anisimova et al. 2011) as the measure of support for each branch. Figure 3 shows the results of this analysis applied to amino acid sequences. As expected, filtering consistently led to an increase in the false negative rate for all conditions. This is consistent with our other observations, which indicate that many phylogenetically informative sites are lost to alignment filtering. Worryingly, the false positive rate also increased in many combinations, particularly when we used lower minimum support thresholds (0.75 and 0.9) in the Fungi and Eukaryote data sets. With more stringent thresholds (0.95 and 0.99), the impact of filtering on the false positive rate was less pronounced but in many cases still detrimental. Only in the Bacteria data set did filtering lead to a decrease in the false positive rate. These observations also broadly hold for the nucleotide alignments (Supplementary Fig. 11 available on Dryad at http://dx.doi.org/10.5061/dryad.pc5j0).

### Controls: Alignment and Tree Inference Methods; Effect of Sequence Lengths, Divergence, and Gappiness; Test Assumptions and Evaluation Measures

The findings above were confirmed with a broad range of different controls. First, we assessed the influence of the alignment software on our analyses by repeating them with Mafft (Katoh et al. 2005), ClustalW (Thompson et al. 1994), and T-Coffee (Notredame et al. 2000) (see section “Methods”). For amino acid alignments, the effect of filtering is practically the same for all alignment methods (Supplementary Fig. 12 available on Dryad at http://dx.doi.org/10.5061/dryad.pc5j0). For DNA alignments, we observed more variation in the effect of filtering among aligners, but the general trends are unchanged: in the overwhelming majority of the combinations, filtering worsens the trees obtained (Supplementary Fig. 13 available on Dryad at http://dx.doi.org/10.5061/dryad.pc5j0). The single instance of a significant improvement was obtained with T-Coffee (two-sided Wilcoxon test, α=0.01), but T-Coffee has been shown to perform poorly on DNA sequences (Dessimoz and Gil 2010) and hence its baseline is worse than that of other methods (Supplementary Fig. 13 available on Dryad at http://dx.doi.org/10.5061/dryad.pc5j0). As the version of T-Coffee used in this study is several releases behind the latest version, we also repeated this particular analysis with a more recent version (v.10.00). The results we obtained were consistent with our previous observations (Supplementary Fig. 14 available on Dryad at http://dx.doi.org/10.5061/dryad.pc5j0). Based on that, we decided not to recompute all other results with that newer version of T-Coffee.

Second, to assess the influence of the tree building method on our analyses, we compared the results using maximum likelihood trees with those using weighted least-squares distance trees (Gonnet et al. 2000). For both amino acid (Supplementary Fig. 15 available on Dryad at http://dx.doi.org/10.5061/dryad.pc5j0) and nucleotide data (Supplementary Fig. 16 available on Dryad at http://dx.doi.org/10.5061/dryad.pc5j0), the effect of alignment filtering with distance trees remained overwhelmingly detrimental on average, though less so than with maximum likelihood trees.

Third, to ensure that our observations hold for a broad range of sequence length, sequence divergence, and “gappiness”, we repeated the analyses on data partitioned into quartiles according to these aspects. Average length could conceivably affect the analysis, because under typical models of sequence evolution, confidence intervals may be expected to tighten with the square root of the alignment length. Thus, removing for instance half of the sites can be expected to introduce more error in trees inferred from shorter alignments than in trees inferred from longer alignments. However, our empirical results suggest that filtering remains detrimental on average even for the longest quartile (Supplementary Figs. 17 and 18 available on Dryad at http://dx.doi.org/10.5061/dryad.pc5j0).

Likewise, one could imagine a different impact of filtering for different average sequence divergence, because highly variable sites can be signs of alignment error or sequence artifacts. Yet for all three phyla, filtered alignment resulted in poorer trees for all observed evolutionary ranges (Supplementary Figs. 19 and 20 available on Dryad at http://dx.doi.org/10.5061/dryad.pc5j0).

Furthermore, to investigate whether filtering showed differential performance depending on the “gappiness” of the alignments, we partitioned the data according to the proportion of gaps in the original alignments. Once again, filtering proved detrimental across all bins (Supplementary Figs. 21 and 22 available on Dryad at http://dx.doi.org/10.5061/dryad.pc5j0). Even in the data quartiles with highest gap proportion, we found many instances where filtering led to significantly poorer trees.

Fourth, to control for our choice of branch support measure in Figure 3, we repeated the analysis using branch length as a crude but very different measure of branch support. By treating branches of length 0.1, 1, 2, and 5 PAM (the average number of mutations per 100 amino acids) units as unresolved, we confirmed the observation that both negative and false positive rates tend to worsen after filtering (Supplementary Fig. 23 available on Dryad at http://dx.doi.org/10.5061/dryad.pc5j0).

So far, all results report performance in terms of the mean Robinson–Foulds (RF) distance between reconstructed and reference tree. As alternative evaluation measures, we also computed the fraction of trees where the topology improved, remained constant, and worsened in terms of RF distance (Supplementary Fig. 24 and 25 available on Dryad at http://dx.doi.org/10.5061/dryad.pc5j0). In the majority of cases, the topology remained unchanged. When it changed, in 25 out of the 30 method data set combinations, changes were overwhelmingly for the worse. In four cases there was practically no difference between the number of topologies improved or worsened. In only one combination—Noisy on the bacteria data set—a slight majority of the changes were toward better topologies.

Sixth, to ensure that the results are not confounded by the enrichment approach, which adds portions of homologous sequences based on local alignments (Figure 1, see section “Methods”), we also tested enrichment using global alignments–i.e. entire protein sequences. Indeed, since local alignments are inherently limited to the best matching portion, filtering methods might conceivably have more opportunities to perform well on globally enriched sequence sets. However, there was no material difference between the two (Supplementary Fig. 26 available on Dryad at http://dx.doi.org/10.5061/dryad.pc5j0), which indicates that this choice of enrichment strategy has a negligible, if any, effect on our assessments.

As a seventh set of controls, we repeated the analyses using a different criterion of alignment quality altogether: the minimum duplication test, described above, which assesses the number of gene duplications in a gene family (Dessimoz and Gil 2010). The minimum duplication test is based on two separate data sets each containing instances of 30–60 homologous sequences drawn from 18 animal genomes and 18 fungi genomes respectively (see Supplementary Materials available on Dryad at http://dx.doi.org/10.5061/dryad.pc5j0 for a list of species and key statistics of the two data sets). Recall that if a filtering method reduces the number of implied duplication nodes on average, it is considered to be beneficial. Conversely, if a filter increases the number of duplications on average, it is considered to be detrimental. Under this test, the difference between unfiltered and filtered alignment showed in all cases an increase in gene duplication and therefore a worsening of the trees upon filtering; in about half of the cases, this was statistically significant (Supplementary Fig. 27 and 28 available on Dryad at http://dx.doi.org/10.5061/dryad.pc5j0).

### Impact of Alignment Filtering on the Ensembl Compara Pipeline and Data

In light of the overall negative effect of alignment filtering on phylogenetic inference under all considered conditions, and to minimize the risk of systematic errors in our data sets or methods, we replicated the investigation in an independent study. This was done on a separate data set, using a different criterion of accuracy, and implemented in separate computer programs. We assessed the impact of alignment filtering on the Ensembl Compara pipeline (Vilella et al. 2009), which infers reconciled gene and species phylogenies as part of the Ensembl database (Flicek et al. 2013). In its usual setup, the pipeline filters alignments moderately prior to tree reconstruction, using ClustalW's column score (required minimum score: 11).

We investigated the effect of alignment filtering on the average number of gene losses per branch using selected filtering methods (TrimAl, Noisy, BMGE, Guidance, ClustalW's column score, removal of random columns, removal of columns with most gaps).

**Figure 4.**
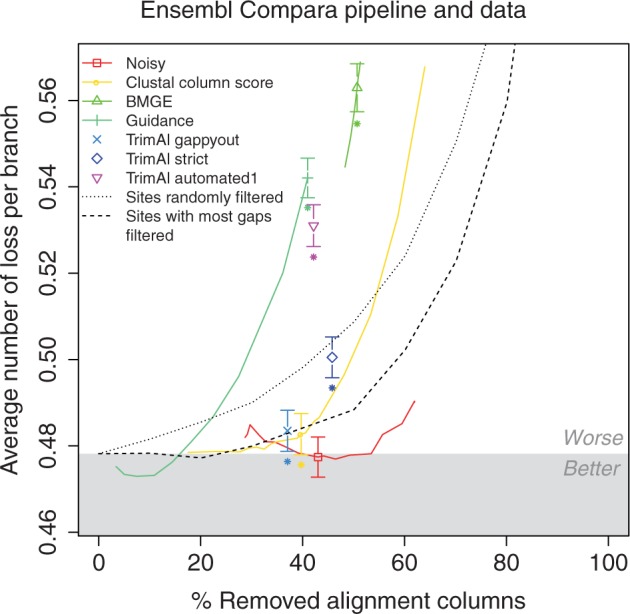
Reanalysis on Ensembl Compara confirms main findings. Points correspond to filtering methods under default parameters. Filtered alignments improving over unfiltered alignment fall in the gray region. The two dotted lines correspond to results obtained with two simplistic filtering methods (see main text). Colored lines are linear interpolations between additional points obtained with non-default parameters and correspond to results obtained by varying the parameters of filtering methods (not available for TrimAl). If a filtering method with default parameters yields significantly different (two-sided Wilcoxon test, α=0.01) results than unfiltered alignments, a star is displayed below the corresponding point.

As Fig. 4 shows, these separate analyses corroborated all the main findings above: that alignment filtering does not improve Ensembl trees over unfiltered alignments; that this remains true even after parameter optimization (for Noisy, ClustalW, BMGE, Guidance, and the simple baselines); that in most conditions, filtering methods did not significantly outperform simple baselines; and that trees tended to get worse as the strength of filtering increased.

Because of the broad distribution of gene tree sizes (i.e., number of genes per tree) in Ensembl (Supplementary Fig. 5 available on Dryad at http://dx.doi.org/10.5061/dryad.pc5j0), we also sought to determine the impact of problem size on the effectiveness of alignment filtering. We separately reassessed the impact of filtering for small, medium, and large data sets (see section “Methods” for exact definitions). Although our main conclusions still hold, we observed a few cases for which filtering yielded a small but significant improvement (Supplementary Fig. 29–31 available on Dryad at http://dx.doi.org/10.5061/dryad.pc5j0). Consistent with our previous observations, light filtering with Guidance yielded a marginal improvement. The other few significant cases of improvement were concentrated in the small data sets, which generally showed irregular results (as indicated by jagged performance curves, (Supplementary Fig. 29, top left available on Dryad at http://dx.doi.org/10.5061/dryad.pc5j0) and very high levels of tree discordance. In contrast, filtering on medium and large data sets led to stronger and consistent worsening of the trees when 20% or more of sites were removed.

### Simulation Study

To gain a better understanding on the impact of filtering on tree inference accuracy, we also repeated our analyses on simulated data. We generated reference alignments of 30 sequences using the software ALF (Dalquen et al. 2012; see section “Methods” for details) and assessed filtering using the species discordance test, using true trees as reference.

**Figure 5.**
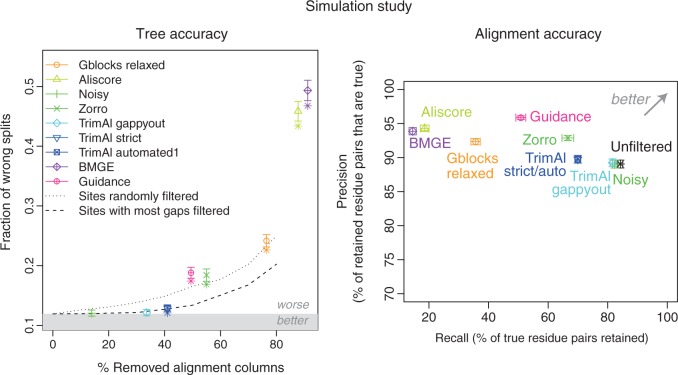
Effect of alignment filtering on simulated data (500 alignments with 30 sequences each): induced tree and alignment accuracy. Tree accuracy (left): the measure of error is the average RF distance between the reference trees and trees reconstructed from Prank + F alignments filtered by the various approaches. Trees were reconstructed using PhyML. Filtered alignments improving over unfiltered alignment fall in the grey region. The two dotted lines correspond to results obtained with two simplistic filtering methods (see main text). Points correspond to default parameters. If a filtering method with default parameters yields significantly different (two-sided Wilcoxon test, α=0.01) results from unfiltered alignments, a star is displayed below the corresponding point. Error bars indicate the standard error of the mean. Alignment accuracy (right): precision and recall for the various filtering methods, using sum-of-pair scoring function (see section “Methods”).

Results on simulated data were consistent with our findings on empirical data. Filtering did not lead to better trees on average (Fig. 5, left). Likewise, though parameter optimization improved the performance of the methods, filtering remained generally counterproductive. Also, filtering methods performed broadly in line with simple baseline methods, with more filtering yielding poorer results.

One advantage of simulated data is that the true alignments are known with certainty. We used this knowledge to directly assess the impact of filtering on alignment quality, in terms of precision (fraction of residue pairs in the filtered alignment that are truly homologous) and recall (fraction of homologous pairs that are present in the filtered alignment). Ideally, filtering should increase precision while maintaining recall, but instead with most filtering methods, precision only moderately improved while recall substantially dropped (Fig. 5, right). This remains true if we use column-wise instead of pairwise measures of alignment accuracy (Supplementary Fig. 32 available on Dryad at http://dx.doi.org/10.5061/dryad.pc5j0).

One concern with simulation is whether the choice of parameter yields data sets that are representative of real data. Our simulated data depart in many ways from real data but we nevertheless note that they result in 90% of pairwise residues correctly aligned (Fig. 5, right), which is in line with that used in other empirical benchmarks (Subramanian et al. 2008). We also repeated the analyses on a less divergent simulated data set and obtained consistent results (Supplementary Fig. 33 available on Dryad at http://dx.doi.org/10.5061/dryad.pc5j0).

## Discussion

Altogether, the picture that emerges from the combination of the different empirical tests and data sets coherently indicates that current alignment filtering methods do not generally lead to better trees. On the contrary, there were many instances where filtering worsened the trees significantly. Furthermore, the few combinations of methods, data, and parameters for which we observed an improvement revealed no clear patterns, making it difficult to predict when existing filtering methods can be effective. Even for these positive cases, the improvement was modest.

The empirical tests used in this study are based on assumptions that might be violated in individual problem instances but these are unlikely to have a strong influence on the aggregate results. For instance, what happens to the species discordance test if some of the input sequences are not true orthologs? In instances where orthology inference is erroneous, an improvement due to filtering can potentially be misreported as worsening. Conversely, a worsening can potentially be misreported as an improvement. However, as long as errors in ortholog calling are not correlated with the performance of individual filtering strategies, the overall ranking of the methods will remain unaffected. Likewise, while the parsimony assumption might not hold for gene families with rampant duplications and losses, the minimum duplication test gives meaningful results as long as the errors thereby introduced are not biased in favor of a particular filtering method.

Our results on empirical data are also backed by simulated data. The strong agreement between the two types of analysis was not a given, because simulated data tend to be easier to model than real data and therefore filtering on the former could have been expected to fare better than on the latter. It nevertheless indicates that our findings hold across quite different data sets.

Filtered alignments led to poorer trees for different combinations of data sets, aligners, tree reconstruction methods, and evaluation criteria. Furthermore, these results held for a considerable range of sequence lengths, divergences, and gap proportions. In particular, it is perhaps surprising that on highly diverged—thus hard to align—sequences, filtering does not appear to do particularly well. We hypothesize that tree inference might be robust to alignment errors between highly divergent sequences (or subsequences) but at the same time sensitive to loss of any genuine phylogenetic information incurred by excessive filtering. More work is needed to test the validity of this explanation.

One reason for the poor performance of current filtering methods appears to be that they remove columns too aggressively; in particular, this is the case for Gblocks and BMGE with default parameters, which appear to have an excessively low tolerance for columns containing gaps. Generally, the methods that removed more columns performed worse. Correspondingly, the best parameters values we found for Noisy and Zorro were those resulting in the fewest removed alignment columns. In part, this may be explained by the substantial phylogenetic signal that may be contained in sparse (gap-rich) regions, previously discussed in the context of both multilocus (Thomson and Shaffer 2010; Roure et al. 2013) and single-gene phylogenies (Dessimoz and Gil 2010).

Some solace lies in the observation that modest amounts of filtering have little impact on the reconstructed trees but decrease computational time, which typically scales linearly with alignment length. For instance, in the Ensembl pipeline, a ClustalW minimum column score of 3 decreases the computation burden by 25% with no apparent worsening of the trees. In other contexts, appropriate cutoff values need to be carefully established as they will depend on the particularities of individual data sets. Our results suggest that filtering up to 20% of alignment positions is relatively safe.

We finish this section by discussing caveats. First, our empirical tests were limited to single-locus alignments. As such, we could not investigate the effect of filtering concatenated alignments. However, the lack of positive trend with respect to sequence length when filtering single-gene alignments (Supplementary Fig. 17–18 available on Dryad at http://dx.doi.org/10.5061/dryad.pc5j0) does not bode well for filtering concatenated alignments. Second, this study focused on tree topology and ignored the problem of inferring accurate branch lengths. This is mainly due to the limitations of our empirical tests, which can only assess topology. However, there are also inherent complications in assessing branch length accuracy when part of the data gets removed. For instance, if the fast evolving sites are preferentially filtered out, the *true* branch length (in average number of substitution per site) gets shorter, thus making the true length a moving target. Third, the number of sequences contained in most alignments considered here was relatively low by current standards, with about 30 sequences on average in the species discordance and minimum duplication tests. However, the Ensembl Compara data set contained larger alignments, with approximately 20% of alignments containing more than 100 sequences. The filtering performance on these larger alignments was below average (Supplementary Fig. 29 available on Dryad at http://dx.doi.org/10.5061/dryad.pc5j0), but due to the risk of latent confounders in these larger gene families (recall that the number of sequences in Ensembl Compara families is not a randomised controlled trial), we cannot draw strong conclusions from this particular result. Fourth, the automated nature of our test pipelines is not fully representative of typical phylogenetic studies, which are often restricted to relatively well-established markers and often involve manual intervention (View 2009). Fifth, although this is to our knowledge the most extensive comparative study of filtering methods to date, we necessarily had to leave out some methods and approaches. In particular, we did not consider filtering strategies that remove individual sequences or characters. How these perform remains to be investigated. And sixth, we disclose that in spite of the several lines of evidence and numerous controls provided in this study, one anonymous referee remained skeptical of our conclusions. His/her arguments were: (i) instead of using default parameters or globally optimized ones, filtering parameters should be adjusted for each data set; (ii) the observations that, in some cases, phylogenies reconstructed using a least-squares distance method were more accurate than phylogenies reconstructed using a maximum likelihood method (Supplementary Figs. 7–10 available on Dryad at http://dx.doi.org/10.5061/dryad.pc5j0), and that ClustalW performed “surprisingly well” compared with other aligners, are indicative that the data sets used for the species discordance test are flawed; (iii) the parsimony criterion underlying the minimum duplication test and the Ensembl analyses is questionable.

## Outlook

For filtering to be worthwhile, the decrease in phylogenetic noise achieved should exceed the loss of phylogenetic signal incurred. Our results indicate that with current filtering methods, this is generally not the case. However, this does not necessarily imply that filtering is inherently a bad idea. By providing new methods to systematically and rigorously test the impact of filtering on tree reconstruction, we hope that this study will help lay the methodological foundations to guide the development of better filtering methods.

Whether effective filtering methods will eventually come to fruition is yet to be seen. But from the methodology standpoint, filtering is a “band-aid” solution to the deeper problem of handling uncertainty in the alignment. More statistically sound approaches have been developed, which ascribe probabilities to alignment columns (Lunter et al. 2008; Bradley et al. 2009) or compute a distribution of alignments (Suchard and Redelings 2006; Novák et al. 2008) and use this information in the tree-building phase. These methods are computationally demanding, which limits their field of application in practice, but one can hope that they will prove to be more effective at dealing with alignment uncertainty. Computational resources permitting, we will be looking into them next.

## Supplementary Material

Data available from the Dryad Digital Repository: http://dx.doi.org/10.5061/dryad.pc5j0.
